# 苯丙胺类、氯胺酮、卡西酮类毒品手性分离的研究进展

**DOI:** 10.3724/SP.J.1123.2020.05020

**Published:** 2021-03-08

**Authors:** Wenchuan TANG, Jing CHANG, Yuanfeng WANG, Aihua WANG, Ruihua WANG

**Affiliations:** 1.公安部物证鉴定中心, 北京 100038; 1. Public Security Department Material Identification Center, Beijing 100038, China; 2.中国政法大学证据科学研究院, 北京 100088; 2. Institute of Evidence Law and Forensic Science, China University of Political Science and Law, Beijing 100088, China

**Keywords:** 手性分离, 对映异构体, 毒品, 苯丙胺类, 氯胺酮, 卡西酮类, 法医毒物学, chiral separation, enantiomer, drugs, amphetamines, ketamine, cathinones, forensic toxicology

## Abstract

对映异构体在自然界中普遍存在,在药物化学领域尤为突出。虽然手性药物的对映异构体之间具有相同的化学结构,但它们在药理、毒理、药代动力学、代谢等生物活性方面存在明显差异。苯丙胺类、氯胺酮、卡西酮类毒品也是如此,这3类毒品的手性分离研究在常见毒品中具有代表性。目前常用的手性分离色谱方法有气相色谱法(GC)、高效液相色谱法(HPLC)和毛细管电泳法(CE)。苯丙胺类、氯胺酮、卡西酮类毒品使用以上3种方法进行的手性分离研究具有一定共性:GC较多使用*N*-三氟乙酰-L-脯胺酰氯和(+)*R*-*α*-甲氧基*α*-三氟甲基苯乙酸两种典型的手性衍生化试剂,HPLC主要应用蛋白质类、多聚糖类和大环抗生素类3种手性固定相,CE中环糊精及其衍生物是最常用的手性选择剂。然而这3种手性分离方法存在各自的不足,GC存在手性衍生化引入杂质、反应温度高影响手性分离等问题,HPLC的应用范围比较有限,成本较高,CE没有明确的方法判断哪种物质是合适的手性选择剂。近年来,这3类毒品的手性分离研究在法医毒物学领域的应用有各自的特点,苯丙胺类毒品的手性分离研究多用于推断市场上毒品的原型及合成路线,氯胺酮的手性分离研究涉及多种生物检材,卡西酮类毒品侧重于手性分离方法的广泛适用性。该文主要遴选近10年国内外核心期刊的文献,对苯丙胺类、氯胺酮、卡西酮类毒品的手性异构体特点及色谱法的手性识别机理进行简单介绍,重点对已有研究的共性以及手性分离在法医毒物学中的应用等内容进行综述。基于以上研究,该文提出未来可以从以下3个方面进行深入研究:一是利用计算机技术建立分子模型深入探究手性识别机理;二是研发新型技术,对超临界流体法进行商用研究;三是将手性分离应用于司法实践、医药研发等实际工作领域。

## Abstract (en)

Enantiomers are ubiquitous in nature, and they are especially important in the field of pharmaceutical chemistry. Although the enantiomers of chiral drugs have identical chemical structures, they differ notably in their pharmacological, toxicological, pharmacokinetic, metabolic, and other biological activities. The same is true for amphetamines, ketamine, and cathinones, as the chiral separation of these three drugs is representative of drugs. Gas chromatography (GC), high performance liquid chromatography (HPLC), and capillary electrophoresis (CE) are widely used for the chiral separation of these three kinds of drugs. There are some similarities among the three methods for the chiral separation of amphetamines, ketamine, and cathinones: *n*-trifluoroacetyl-L-prolinyl chloride and (+)*R*-α-methoxy-α-trifluoromethylphenylacetic acid are the two typical chiral derivatization reagents used in GC. In HPLC, three kinds of chiral stationary phases are used: proteins, polysaccharides, and macrocyclic antibiotics. Cyclodextrin and its derivatives are most commonly used in CE. However, these three methods have inherent shortcomings. In the case of GC, impurities produced during chiral derivatization may interfere with the analysis, and high reaction temperatures affect the efficiency of chiral separation. HPLC has limited application scope and is expensive. In CE, there has no established process to determine the appropriate chiral selector. In recent years, research into application of the chiral separation of the above-mentioned three kinds of drugs has its own characteristics in forensic toxicology. The chiral separation of amphetamine drugs is mostly used to infer the prototype and synthesis route of drugs on the market. The chiral separation of ketamine involves a variety of biological samples. For cathinones, chiral separation methods emphasize their wide applicability. In this review, 66 reports published in professional local and overseas magazines during the past decade are collated. The characteristics of the enantiomers of amphetamines, ketamine, and cathinones as well as the mechanism of chiral recognition are briefly introduced. The commonness of the research and the application of chiral separation in forensic toxicology are reviewed. This paper proposes that the chiral separation of drugs can be further investigated from the following three aspects: 1) the use of computer technology to establish a molecular model for exploring the mechanism of chiral recognition; 2) developing new technologies for chiral separation and carrying out commercial research on the supercritical fluid method; 3) applying chiral separation to judicial practice, pharmaceutical research and development, and other practical fields.

两个互为镜像而不能重合的立体异构体,称为对映异构体。对映异构体的分子中有一个连有4个不同原子或基团的碳原子,叫手性碳原子。对映异构体在自然界中普遍存在,并且在生命活动中起到极为重要的作用,例如一些氨基酸和糖类对人体有营养作用,但其对映异构体没有营养价值,甚至对人体会产生副作用。在药物化学领域,对映异构体的存在也是极为常见的,它们具有极为相似的理化性质,但是在人体中的作用部位、毒性、代谢过程等方面都存在较为明显的差异。部分毒品也是如此,例如,对于甲基苯丙胺而言,*S*-甲基苯丙胺是常见的毒品,主要作用于中枢神经系统,而其对映异构体*R*-甲基苯丙胺则主要作用于心血管系统,多用于临床医疗^[1]^。因此,正确识别、恰当分离有旋光性的毒品及其对映异构体,不仅有利于司法领域认定对毒品犯罪的准确定罪、量刑,而且对医药、科研领域也有所裨益。

针对我国司法实践,本文重点对苯丙胺类、氯胺酮、卡西酮类3类常见毒品的手性色谱分离方法的共性以及手性分离在法医毒物学中的应用等研究进展进行综述,以期对毒品犯罪的司法鉴定工作、相关医药研究提供参考。

## 1 3类毒品对映异构体的特点

### 1.1 苯丙胺类

苯丙胺类兴奋剂主要以兴奋和致幻作用为主,其可以选择性地作用于脑干以上的中枢神经系统,通过使中枢和外周单胺类神经递质水平升高,增强中枢神经系统活动,间接产生药理和毒理学作用^[2]^。苯丙胺类兴奋剂为苯丙胺(AM)的衍生物,结构通式见图1a。

**图1:**
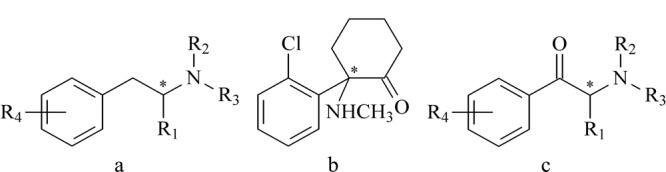
(a)苯丙胺类兴奋剂、(b)氯胺酮和(c)卡西酮类毒品的结构式

除了AM外,苯丙胺类兴奋剂还包括甲基苯丙胺(冰毒,MAMP)、3,4-亚甲二氧基苯丙胺(MDA)、3,4-亚甲二氧基甲基苯丙胺(MDMA)、3,4-亚甲二氧基乙基苯丙胺(MDEA)、3-甲氧基-4,5-亚甲二氧基甲基苯丙胺(MMDMA)、二甲氧基乙基安非他明(DOET)等。《2018年中国毒品形势报告》表明,“在240.4万名现有吸毒人员中,滥用冰毒人员135万名,占56.1%,冰毒已取代海洛因成为我国滥用人数最多的毒品”^[3]^。

麻黄素、伪麻黄素是合成苯丙胺类毒品的重要原料,这两种分子均具有手性碳原子。在合成过程中,麻黄素、伪麻黄素的手性碳原子不参与反应,因而利用这两种原料合成的苯丙胺类毒品是对映异构体^[4]^。苯丙胺类的对映异构体在药效性、毒性、代谢等方面有较大差异。以MAMP为例,*R*-MAMP多用于医疗过程,被用作鼻腔减充血药物的活性成分,而*S*-MAMP因其成瘾性和滥用趋势被界定为中枢神经系统的强效兴奋剂^[5]^;相比*R*-MAMP,等剂量的*S*-MAMP对人体心血管的作用更强^[6]^;当摄入外消旋体MAMP 16 h后,可以检测出*S*-MAMP在体内代谢程度更低^[7]^,由此可见,*S*-MAMP更容易对人体产生长时间作用。

### 1.2 氯胺酮

氯胺酮是一种被列管的精神药品。它具有一定的致幻作用和成瘾性,在人体内的作用机制较复杂,能选择性地阻断痛觉冲动向丘脑新皮层系统的传导,兴奋脑干及边缘系统,引起意识模糊、短暂性记忆缺失,产生梦幻、肌张力增加、血压上升等症状^[8]^。氯胺酮在我国滥用的情况较为严重。根据《2018年中国毒品形势报告》,我国滥用氯胺酮的人数仅次于冰毒和海洛因。氯胺酮的化学名称是2-氯苯-2-甲基胺环己酮,分子中有一个手性碳原子,结构式见图1b。氯胺酮早在1970年就被作为麻醉剂用于临床,临床使用的一般为等量*R*-氯胺酮和*S*-氯胺酮的外消旋体混合物。两种氯胺酮对映异构体的麻醉效果有所差异,*S*-氯胺酮的麻醉效果是*R*-氯胺酮和外消旋体氯胺酮的2~5倍^[9]^,相比外消旋体和*R*-氯胺酮,使用*S*-氯胺酮作为麻醉剂时,产生的副作用更小^[10]^。依据以上特点和持续的临床研究,近年来一些国家已将*S*-氯胺酮作为临床用外消旋体氯胺酮的替代品。此外,近年来氯胺酮的抗抑郁作用引发了科学家们的关注,*R*-氯胺酮相比*S*-氯胺酮在抗抑郁药物领域更有潜力^[11]^。

### 1.3 卡西酮类

卡西酮类毒品具有很强的兴奋作用。近些年来,卡西酮类毒品在毒品市场中越来越泛滥,尤其是娱乐场所。国内外因滥用卡西酮类毒品引发的中毒、致死案例越来越多^[12]^。卡西酮是卡西酮类毒品中最为主要的一种,它也被称为苯甲酰乙胺或*β*-酮苯丙胺,是一种单胺类生物碱,存在于阿拉伯茶中,其化学性质与麻黄碱和其他苯丙胺类物质类似。卡西酮的结构与AM相似,包含一个手性碳原子,但又不同于AM,它有一个酮官能团。卡西酮类毒品是以卡西酮的分子结构为基础而衍生出来的一类毒品,结构通式见图1c。其他常见的卡西酮类毒品有甲卡西酮、4-甲基甲卡西酮(4-MMC)、3,4-亚甲基二氧吡咯戊酮(MDPV)、4-氟甲卡西酮(4-FMC)、3-氟甲卡西酮(3-FMC)、3,4-二甲基甲卡西酮(3,4-DMMC)等。卡西酮类毒品在药理作用上与苯丙胺类毒品相似,二者在构型上的特点也相似,即*S*构型比*R*构型对中枢神经系统的作用强^[13]^。甲卡西酮是卡西酮类毒品中常见的一种,是卡西酮的*N*-甲基衍生物,比卡西酮的药效强数倍。Glennon等^[14]^通过小鼠实验证实,*S*-甲卡西酮和*R*-甲卡西酮对中枢神经系统都有刺激作用;*S*-甲卡西酮药效是*R*-甲卡西酮药效的3~5倍,是*S*-苯丙胺药效的2倍。

## 2 3类毒品对映异构体的手性分离

### 2.1 色谱法分离对映异构体的原理

对映异构体的手性分离方法主要分为色谱法和非色谱法两大类。由于非色谱法不能获得较纯的对映异构体,并且操作烦琐,耗时较长,现已被色谱法逐渐取代。色谱法分离对映异构体的原理主要基于Dalgliesh提出的“三点手性识别模式”^[15]^, Pirkle等^[16]^发展了“三点手性识别模式”,并较为详尽地阐释了对映异构体的拆分原理,在对映异构体和手性作用物之间存在至少3个相互作用点,其中至少一个作用点应为立体化学作用,另外两个作用点可以是不同类型的作用力,例如氢键、偶极相互作用、电荷转移、配位化合物、位阻排斥、疏水吸引等分子间作用。两个对映异构体与手性作用物之间形成非对映体络合物的稳定性有差异而得到分离。

如图2所示,手性作用物a与一种待分离的对映异构体b形成了3组相互作用点,即A与A'、B与B'和C与C' 3个作用力。然而,手性作用物a与另一种待分离的对映异构体c之间不存在C与C'作用力,因此a与b、a与c形成的非对映分子络合物的性质不同,可以通过色谱进行分离。

**图2:**
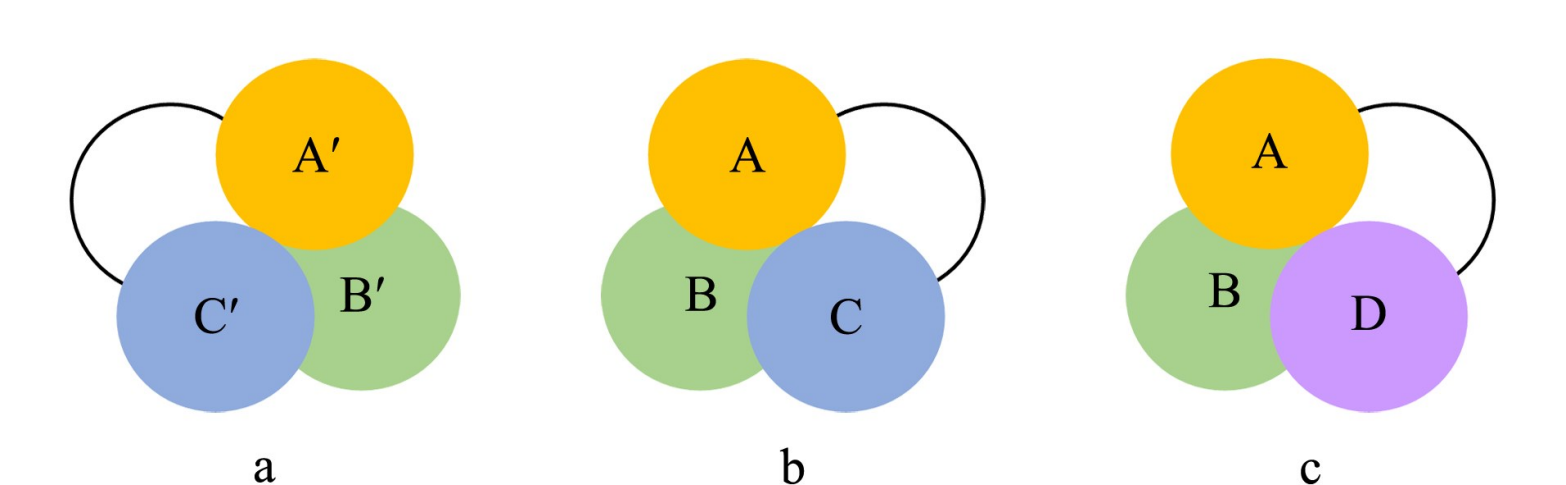
“三点手性识别模式”示意图

然而,随着色谱法中手性固定相的不断发展,一些较复杂的手性固定相与待分离的对映异构体之间的手性识别机理不能完全用“三点手性识别模式”解释。随着研究和认识的不断深入以及科技的快速发展,近年来关于手性识别机理的研究方法主要有热力学分析法^[17,18,19,20]^,通过计算机建立分子模型模拟手性识别方法^[21,22,23,24]^,核磁共振、荧光分析等光谱学法^[25,26]^等。

### 2.2 常见的色谱分析方法

常见的手性药物分离和检测方法有气相色谱法(GC)、高效液相色谱法(HPLC)、毛细管电泳法(CE)、超临界流体色谱法(SFC)等分离技术,以及上述分离技术和质谱(MS)、紫外光谱(UV)等检测技术的联用。苯丙胺类、卡西酮类对映异构体的色谱分离方法集中使用GC、HPLC和CE,氯胺酮对映异构体的色谱分离则大部分使用HPLC和CE。

2.2.1 GC

GC是色谱法中发展较早的一项检测技术,也是较早应用于对映异构体分离的检测技术,其特点是对映异构体的分离度、重现性和精度都很高。在具有挥发性和热稳定性的手性分子分离方面,GC展现出明显的优势。

早期苯丙胺类、甲卡西酮类毒品对映异构体使用GC分离的较多,其特点是进样分析前需使用手性衍生化试剂,这两类毒品常用的手性衍生化试剂主要有*N*-三氟乙酰-L-脯胺酰氯(L-TPC)和(+)*R*-*α*-甲氧基*α*-三氟甲基苯乙酸(MTPA)。最初在苯丙胺类、甲卡西酮类毒品的手性分离中,使用L-TPC作为手性衍生化试剂的情况较为普遍,因为酰氯的反应性最强,与仲胺、仲羟基也能发生反应,但由于L-TPC商品化纯度的影响,以及在存放过程中其会发生消旋化,生成一定比例的另一构型对映异构体,该杂质会影响衍生化产物的纯度,使得检测结果产生8%~19%的偏差^[27]^。而MTPA的衍生化效果比L-TPC更好,孟品佳^[28]^对比了L-TPC和MTPA对苯丙胺类毒品对映异构体的分离情况,使用MTPA进行衍生化产物的稳定性明显好于L-TPC衍生化产物,并且可以对苯丙胺类毒品的对映异构体进行定量分析^[29]^。卡西酮类毒品对映异构体也能够使用L-TPC进行手性衍生化,但L-TPC的外消旋化会对部分卡西酮类毒品的手性分离产生影响,Mohr等^[30]^的研究表明,使用L-TPC进行衍生化会使*S*-甲卡西酮转化成*R*-甲卡西酮。与使用L-TPC进行手性衍生化相比,卡西酮类毒品经MTPA衍生化后的产物有更好的分离度,能够在质谱中获得较优的离子对^[31]^。

GC技术是开发得较早的一种分离对映异构体的色谱技术,但其存在一些固有的局限性,例如:进样前需进行手性衍生化,使得操作较复杂;操作温度相对比较高,使衍生化后的非对映异构体之间的相互作用能差别变小,对映异构体分离困难^[32]^;柱温高导致对映异构体的选择性降低等。由于GC技术进行手性分离时必须使用手性衍生化试剂,手性衍生化试剂的稳定性非常重要,如果其中一种对映异构体会对手性衍生化试剂产生消旋化的影响,这将导致两种对映异构体衍生化生成的非对映异构体出现混淆,从而影响对映异构体的正确分离检测,比如氯胺酮不宜使用常见的脯氨酰类手性衍生化试剂进行衍生就是这个原因^[33]^。这些局限性导致一部分对映异构体不宜通过GC技术实现分离,目前使用该技术进行手性分离的研究逐渐减少。

2.2.2 HPLC

HPLC是在GC的基础上发展起来的一项色谱技术,是手性分离领域应用最广泛的色谱方法。相对于GC的局限性,HPLC能够有效避免因温度过高对手性分离产生的影响。HPLC可以分为间接法和直接法,其中,间接法是指手性衍生化试剂法,即目标物经衍生化处理后再使用HPLC分离;直接法是指直接选用手性固定相或者在流动相中加入手性添加剂。近年来,直接法在手性分离中应用较多,适用于多种对映异构体的混合分离,该方法还能避免间接法中因添加手性衍生化试剂引入杂质对分离检测带来的影响。

苯丙胺类、氯胺酮、卡西酮类毒品对映异构体的分离检测广泛使用了HPLC技术,其中现有文献中苯丙胺类毒品对映异构体的分离研究使用手性固定相法的居多,也有部分采用手性添加剂法,而氯胺酮和卡西酮类则大多数使用手性固定相法。该3类毒品对映异构体使用的手性固定相涉及3种不同类型的手性色谱柱,即蛋白质类手性柱、多聚糖类手性柱和大环抗生素类手性柱。蛋白质具有复杂的三维结构,其亚单位L-氨基酸具有手性特异性,因此可以识别对映异构体。由其制成的手性固定相广泛应用于手性药物的识别检测,其优点是可使用水作为流动相且手性选择能力较好。多聚糖类化合物(尤其是纤维素和支链淀粉)本身具有一定的手性识别能力,其衍生后表现出较好的手性识别能力,使其在手性柱方面得到广泛应用。大环抗生素类化合物分子具有立体的环状结构、芳香基团、氨基和羟基等活性基团,包含几个到几十个手性中心。作为手性固定相,大环抗生素类化合物具有手性识别能力强、稳定性高等优点,相体系转化时不发生老化和变性,在对映异构体的分离方面已经得到了较好的应用^[34]^。这3类毒品手性分离研究中,使用较多的手性色谱柱类型、手性选择剂以及使用的流动相总结见表1。

**表1:** 苯丙胺类、氯胺酮、卡西酮类毒品对映异构体研究中常见手性色谱柱类型、手性选择剂及相配的流动相

Type of chiral column	Name of chiral column	Chiral selector	Separated species	Mobile phase	Ref.
Protein chiral column	Chiralpak CBH	cellobiohydrolase	amphetamine derivatives	1 mmol/L ammonium acetate- methanol (85∶15, v/v)	[35]
	Chiralpak AGP	*α*_l_-acid glycoprotein	ketamine	10 mmol/L ammonium acetate- 2-propanol (94∶6, v/v)	[39]
Polysaccharide derivative-based	Chiralpak AS-3R	amylose tris[*S*-*α*-methyl- benzylcarbamate]	ketamine	1 mmol/L ammonium bicarbonate- acetonitrile (54∶46, v/v)	[40]
chiral column	Lux i-cellulose-5	cellulose tris-(3,5-dichloro- phenylcarbamate)	cathinones	*n*-hexane-isopropanol-diethylamine (95∶5∶0.1, v/v/v)	[43]
	ACQUITY UPC2 Trefoil CEL1	cellulose tris-(3,5-dimethyl- phenylcarbamate)	cathinones	*n*-hexane-*n*-butanol-diethylamine (100∶0.3∶0.2, v/v/v) or *n*-hexane- diethylamine (100∶0.2, v/v)	[44]
Macrocyclic antibiotics chiral	Chirobiotic V	vancomycin	ketamine	methanol-acetic acid-triethylamine (100∶0.02∶0.02, v/v/v)	[42]
column	Chirobiotic V2	vancomycin	amphetamine, methamphetamine	20 mmol/L ammonium acetate (0.1% v/v formic acid)-methanol (1∶99, v/v)	[37]

苯丙胺类 使用手性固定相法对苯丙胺类毒品对映异构体进行手性分离的研究中,手性固定相的种类主要是大环抗生素类手性柱和蛋白质类手性柱,多糖类手性柱的表现不佳。Castrignanò等^[35]^对比了Chiralpak CBH、Chirobiotic V和Chirobiotic T 3种手性色谱柱对苯丙胺类毒品对映异构体的分离效果。Chiralpak CBH是蛋白质类手性柱,通常用来拆分碱性化合物的对映异构体,其优势是对包含苯环结构的伯胺和仲胺类化合物的手性分离能力较强。Chirobiotic V和Chirobiotic T是大环抗生素类手性柱,前者的手性选择剂是万古霉素,后者的手性选择剂是替考拉宁,替考拉宁对某些天然和合成氨基酸以及小肽的对映异构体分离能力较为突出^[36]^。3种手性柱中,Chiralpak CBH的总体分离效果略优于Chirobiotic V,而Chirobiotic T可以分离某些ChiralpakCBH不能分离的苯丙胺类对映异构体。该文献^[35]^的方法可以分离废水中AM、MAMP、MDMA等9种苯丙胺类毒品的18种对映异构体,以峰面积对浓度进行线性回归,每种物质线性良好(相关系数(*r*^2^)均大于0.997),方法平均精密度小于10%,仪器平均精密度小于5%。该方法通过监测生物环境中的毒品废物,能够用来跟踪毒品的合成路径和使用路径。张颖怡等^[37]^对比了Chirobiotic V2和Chiralpak IA-3两种手性色谱柱,Chirobiotic V2手性柱是新型键合型Chirobiotic V手性柱,升级后的手性识别能力增强,而Chiralpak IA-3是一种多聚糖类手性柱。实验结果表明:Chirobiotic V2手性色谱柱对胺类化合物立体选择性较优,对AM和MAMP对映异构体的分离效果具有明显优势,可以在12 min内对AM和MAMP的对映异构体实现手性分离,而Chiralpak IA-3手性色谱柱不能分离苯丙胺类对映异构体。

使用手性添加剂法对苯丙胺类毒品进行手性分离时,手性选择剂一般选用环糊精及其衍生物,环糊精及其衍生物加入的体积、流动相的比例会影响手性分离的效果。Taschwer等^[38]^考察了流动相的组成对苯丙胺类毒品对映异构体分离效果的影响。如果流动相是体积比为10∶90的甲醇和水,并加入1%的硫化*β*-环糊精,则保留时间会缩短,但只有AM的对映异构体可以得到分离;如果甲醇和水的体积比变为2.5∶97.5,硫化*β*-环糊精的体积依旧占比1%,则保留时间会增加,但是分离效果仍较差。因此经过优化,流动相的条件最终设置为体积比2.5∶97.5的甲醇和水,硫化*β*-环糊精体积占比2%,成功实现了AM、MDMA等6种苯丙胺类毒品的12种对映异构体的分离。

氯胺酮 氯胺酮对映异构体可以使用蛋白质类手性柱、多聚糖类手性柱和大环抗生素类手性柱进行分离,每种手性柱对应的方法各有特点。

Chiralpak AGP手性柱是蛋白质类手性柱中分离能力最强、使用范围最广的一种,可以对中性、酸性和碱性化合物进行手性分离。使用此种手性柱对氯胺酮对应异构体进行分离的研究始于20世纪90年代初,该色谱柱的分离程度受pH、柱温、有机溶剂比例等色谱条件影响较大,缺点是手性分离时间较长,只能耐受低比例的有机溶剂。蒋娟娟等^[39]^利用该手性柱,建立了检测人体血浆中氯胺酮及其代谢物去甲基氯胺酮对映异构体浓度的方法,并将此方法应用于研究氯胺酮对映异构体的药代动力学。该方法手性分离效果良好,灵敏度高,无基质效应影响,但是进样量不宜超过20 μL,否则会影响峰形。

Chiralpak AS-H是一种多聚糖类手性柱,该手性柱是Chiralpak AGP手性柱升级后的手性柱,其特点是克服了Chiralpak AGP手性柱运行时间长的缺点。Toki等^[40]^利用该手性柱建立了一种快速、高效分离大鼠血浆、大脑和脑脊髓液中氯胺酮对映异构体的方法。该方法的优点除了分析时间短(5 min)外,还有检材用量少,每次仅需5 μL血浆、10 mg脑组织或2.5 μL脑脊髓液,定量限(LOQ)为1 ng/mL(血浆)、0.5 ng/g(大脑组织)和2 ng/mL(脑脊髓液)。多聚糖手性柱CHIRAL-JM-R手性柱可与高比例(有机相体积>30%)的有机试剂乙腈或甲醇一起使用,并显示出与质谱的良好相容性。Li等^[41]^利用该手性柱研究了血浆中氯胺酮对映体的检测方法,以及给狗静脉注射氯胺酮后,两种对映异构体体内药代动力学情况。他们考察了流动相的构成,有机相选择乙腈,常用的甲酸铵、乙酸铵体系不能分离氯胺酮对映异构体,而当水相为碳酸氢铵时,分离效果良好。氯胺酮的两种对映异构体在0.5~500 ng/mL间显示出良好的线性(*r*^2^>0.99), LOQ为0.5 ng/mL,日内精密度小于7.3%,日间精密度小于10%。

选择大环抗生素类手性柱对氯胺酮对映异构体进行分离时,将极性有机流动相模式引入手性分离,能够取得比较好的效果。单丽娜等^[42]^测定人血浆中氯胺酮对映异构体的浓度时,采用Chirobiotic V手性柱,使用的极性有机流动相为甲醇中加入少量有机酸冰醋酸和有机碱三乙胺。该实验探究了冰醋酸和三乙胺比例对手性分离的影响,当冰醋酸和三乙胺在甲醇中的总比例上升时,氯胺酮对映异构体的保留时间和分离度逐渐下降;当甲醇、冰醋酸、三乙胺体积比为100∶0.02∶0.02时,分离效果较佳,可以排除血浆中内源性物质及氯胺酮代谢物对实验结果产生的干扰。

卡西酮类 使用HPLC法分离卡西酮类毒品的对映异构体时,常用手性固定相为多聚糖类手性柱,对流动相的配比要求较高。Kadkhodaei等^[43]^使用Lux i-cellulose-5手性色谱柱,成功实现了47种卡西酮类毒品的94种对映异构体的分离。该实验一开始选用极性有机流动相模式,以乙腈、异丙醇、二乙胺、甲酸(体积比为95∶5∶0.1∶0.1)为流动相,但手性分离结果并不理想,经证实正相模式更优,流动相为正己烷、异丙醇和二乙胺(体积比为95∶5∶0.1)。Hägele等^[44]^以纤维素三-3,5-二甲基苯氨基甲酸酯为手性固定相,建立了一种可以快速分离数十种卡西酮衍生物对映异构体的方法。该实验一开始使用已有文献中的流动相配比,即正己烷、异丙醇、二乙胺的体积比为90∶10∶0.1,调整3种物质的配比为95∶5∶0.1和99∶1∶0.1,但是分离度和保留时间均不佳。之后将异丙醇换成正丁醇,并将配比调整至正己烷、正丁醇、二乙胺体积比100∶0.3∶0.2,可以成功分离33种卡西酮衍生物的66种对映异构体和11种吡咯烷酮衍生物的22种对映异构体。如果将流动相调整为正己烷-二乙胺体积比为100∶0.2,可以拆分另外9种卡西酮衍生物的18种对映异构体和1种吡咯戊酮衍生物的2种对映异构体;该方法还能对其中6种卡西酮衍生物的12种对映异构体混合物进行同时分离。

随着人们对手性识别机理认识的不断深入以及色谱技术的不断发展,HPLC已成为当前手性药物分离检测中最常用和最有效的方法。然而,HPLC也存在一定限制,例如间接法同样存在进样前需进行手性衍生化导致操作较复杂的问题,而直接法则存在手性固定相价格较高的问题。每种手性固定相有其适宜分离的化合物,目前的应用范围有限,即使同属于苯丙胺类、卡西酮类毒品,也会因其他基团的影响而导致需要使用不同种类的手性固定相。很难预测哪种手性固定相适用于具体的某种物质,选择合适的手性固定相通常比较耗时并且成本较高。如果手性固定相改性升级后适用的范围更广,或者有更加准确的手性固定相识别机理,那么有可能解决选择困难和成本高的问题。

2.2.3 CE

CE是一类以毛细管为分离通道、以高压直流电场为驱动力的液相分离方法,其特点是集高效和应用广泛性于一身,相对HPLC技术成本较低,在手性分离方面也显示出了巨大潜力。目前使用CE分离手性化合物的研究中,环糊精(CD)及其衍生物是众多手性选择剂里最常用的一类^[45]^,这在苯丙胺类、氯胺酮和卡西酮类的手性分离研究中也有所体现。这3类毒品使用CE进行手性分离研究的重点主要集中在手性选择剂的种类和浓度对手性分离的影响,使用的手性选择剂主要是环糊精及其衍生物。总体来说,苯丙胺类、氯胺酮、卡西酮类毒品对映异构体使用*β*-CD及其衍生物和硫化*γ*-CD进行分离的效果最佳。

由于*β*-CD的空腔与大部分药物相匹配,且易于制得,因此*β*-CD及其衍生物是电中性环糊精及其衍生物中最为常用的类型之一^[46]^,苯丙胺类、氯胺酮、卡西酮类毒品对映异构体均能使用*β*-CD及其衍生物进行手性分离。Varesio等^[47]^在实验中对比了*α*-CD、*β*-CD和*γ*-CD分离尿液中AM、MDA、MDMA、MDEA外消旋体的效果,结果显示*γ*-CD最不适合苯丙胺类对映异构体的分离,*β*-CD和2-羟丙基-*β*-CD的分离效果较好,相较于*β*-CD, 2-羟丙基-*β*-CD具有更优的特性:稍好一些的溶解性,更短的分析时间以及200 nm下UV检测基线更稳定,因而最终被选定。该实验还比较了2-羟丙基-*β*-CD浓度对手性分离的影响,在10~40 mmol/L的浓度范围中,20 mmol/L是最佳浓度。

硫化*γ*-CD属于阴离子型环糊精,一般认为比中性环糊精的手性分离能力更强,其优势是可以通过静电作用吸附到毛细管壁上,起到类似固定相作用,避免样品吸附,改善分离效果,并且还可以调整对映异构体的出峰顺序。Mikuma等^[48]^以高硫化*γ*-CD为手性选择剂,建立了一种可以分离AM、MAMP、麻黄碱等8种苯丙胺类衍生物的16种对映异构体混合物的方法。Theurillat等^[49]^研究了硫化*γ*-CD分离氯胺酮及其代谢物对映异构体的方法。他们开展了微测定实验,每组实验使用50 μL狗的血浆或血清,将0.6%~0.8%(缓冲液中的体积分数,下同)硫化*γ*-CD作为手性选择剂加入pH=3的100 mmol/L磷酸缓冲液中,于25 ℃的条件下,在16 min内拆分出手性氯胺酮,定量限小于1 ng/mL。实验发现0.66%的硫化*γ*-CD已经可以进行手性分离,硫化*γ*-CD体积分数升高会增加检测时间,体积分数降低会缩短检测时间,体积分数过低可能会导致检测不充分。该方法的实验结果完全可媲美HPLC法。Moini等^[50]^研究了使用CE-MS技术时,高硫化*γ*-CD的体积分数(为0.08%~0.2%)及其在毛细管柱中的填充量对卡西酮类对映异构体分离的影响。当体积分数为0.125%时,手性分离效果最好。基于他们之前的研究,(+)-18-冠-6-四羧酸是一种冠醚类手性选择剂,能对某些氨基酸、神经递质的对映异构体进行分离,将其与CD合用构成二元体系手性分离能力更强。在该实验中,他们将15 mmol/L的(+)-18-冠-6-四羧酸填充到毛细管中,与0.125%高硫化*γ*-CD合用,成功分离了6种卡西酮类衍生物的12种对映异构体。

该3类毒品对映异构体通常使用的是CE技术中最为常见的分离模式,即毛细管区带电泳(CZE),此外也有毛细管电色谱(CEC)模式的研究。CEC模式是CE与液相色谱相结合形成的一种微分离技术,通过在毛细管柱内填充或键合手性固定相,以电渗流为驱动力,根据对映体在手性固定相和流动相之间分配系数及电泳淌度的不同实现分离,该技术在对映异构体的分离研究中有一定优势。Aturki等^[51]^利用CEC建立了一种可以分离10种卡西酮衍生物的20种对映异构体的方法,该方法的核心是毛细管柱中填充直链淀粉-三(5-氯-2-甲基苯氨基甲酸酯)作为手性固定相。电压设置为10 kV,温度为20 ℃,可以在10 min内分离10种对映异构体。与GC、LC相比,CEC具有试剂用量少、手性固定相含量少等优点,因此在对映异构体的分离研究中CEC法具有经济且环境友好的优势。

CE技术是近年来在手性分析中发展最快的技术,已取得重要的地位和得到广泛的应用,但其明显缺点在于没有明确的方法判断哪种物质是合适的手性选择剂,仅停留在经验和实验阶段,各种手性选择剂确切的拆分机制及其影响因素,还有待于进一步研究和探索。目前苯丙胺类、氯胺酮、卡西酮类毒品对映异构体的分离研究中,经常需要对比3种以上的手性选择剂才能选出其中较为合适的一种,如何根据待分离化合物的结构特点判断适合的手性选择剂是需要解决的难点问题。

## 3 3类毒品对映异构体的分离在法医毒物分析中的应用

在法医毒物学领域中,对手性毒品的研究包括对已知毒品对映异构体纯度的检验以及未知毒品性质的认定等方面。建立快速、高效的手性分析方法,对判断毒品来源、认定犯罪嫌疑人罪名、为法官提供量刑参考等方面都有重要影响。

近年来,在法医毒物学分析领域中,苯丙胺类毒品对映异构体的研究主要集中在测定毒品滥用者血浆、毛发、尿液中MAMP和AM的含量,并由此推断市场上毒品的原型及合成路线。Maas等^[52]^通过LC-MS/MS技术,研究了106份由德国两个地区警察提供的案件血浆样本,结果显示在超过99%的检材中,仅检测到*S*-MAMP,从而得出结论,在德国市场上主要流通的是由1*R*,2*S*-麻黄碱或1*S*,2*S*-伪麻黄碱合成的*S*-MAMP,以及外消旋体MAMP。Binz等^[53]^研究了两类人员的头发,第一类是瑞士警方提供的街头服用AM的人员,第二类是服用一种治疗注意缺陷障碍药物(活性成分是*S*-AM)患者,实验发现从第一类人员毛发中检测出的*R/S*-AM对映异构体比例为0.9~1.3,表明这些人员摄入的是外消旋体AM,而除一名患者外,第二类人员的头发种仅检测出*S*-AM。通过对映异构体的比例可以简单判断是服用药物还是摄入AM毒品。Wang等^[54]^通过建立的检测尿液中AM和MAMP对映异构体的方法,成功检测了86份疑似MAMP滥用者的尿样。其中72份样品中只检测到*S*-MAMP和*S*-AM,有14份尿样中两种构型的MAMP和AM都均检出,但*S*-MAMP和*S*-AM占比更大,所有尿样中都没有单独检测出*R*-MAMP阳性,因此可以推断*S*-MAMP是非法滥用药物的主要成分。飞行时间质谱(TOF-MS)可以提供化合物的准确质量信息,因而越来越多地用于筛查未知药物。Cui等^[55]^建立了一种利用毛细管电泳-飞行时间质谱技术筛查苯丙胺类毒品的方法,该方法可以快速、准确地筛查未知样品是否包含AM、MAMP、MDA等9种苯丙胺类毒品的对映异构体。

氯胺酮对映异构体的分离研究涉及的人体相关检材类型非常丰富。Hasan等^[56]^利用液相色谱-串联质谱技术,建立了一种可以从人血清、尿液和排泄物中分离出氯胺酮对映异构体的方法。该方法的优点为所需基质的体积小(200 μL),洗脱液用量少(300 μL),可以延长色谱柱的寿命,并且样品制备速度快,分析时间短且灵敏度更高。该方法还可以分离10种羟基化的氯酮胺代谢物。Porpiglia等^[57]^研究了人毛发中氯胺酮对映异构体的分离方法,他们还通过验证实践中收集到的氯胺酮呈阳性的毛发样品,确认了建立方法的有效性,为法庭科学领域检测毛发中是否含有手性氯胺酮提供了参考方法法。

在过去的几年中,卡西酮类毒品的种类越来越多,针对这个问题,法医毒物学领域的研究趋势是建立的方法能分离数十种卡西酮衍生物的对映异构体,这样的方法可以提高检验效率。Alremeithi等^[58]^通过气相色谱-负化学电离-质谱法(GC-NCI-MS),使用L-TPC进行衍生化,建立了一种可以灵敏分离36种卡西酮衍生物的72种对映异构体的方法。该方法还可以对一次性加入14种卡西酮衍生物的28种对映异构体混合物的尿液和血浆样品进行定量分析。Hägele等^[59]^利用CZE技术建立的方法能分离58种卡西酮衍生物的106种对映异构体,该方法使用*β*-CD、乙酰基-*β*-CD、2-羟丙基-*β*-CD、CM-*β*-CD 4种CD衍生物作为手性选择剂,每种手性选择剂对应不同的工作电压(*β*-CD:阴极30 kV,乙酰基-*β*-CD和2-羟丙基-*β*-CD:阴极29 kV, CM-*β*-CD:阴极22 kV),除工作电压不同外,其他条件一致(10 mmol/L手性选择剂,10 mmol/L磷酸钠,用磷酸调节的pH=2.5,温度25 ℃)。

## 4 结论与展望

分析技术的不断发展对毒品的检测、分离与认定均产生了积极作用。在现有研究中,毒品对映异构体的分离方法主要以GC、HPLC和CE技术为主,另外,近些年还有部分研究使用的是SFC技术,该技术是一种以超临界流体为流动相的色谱分析技术,其特点是既可以分析挥发性差的样品,还具有比HPLC更高的效率,分析时间更短,操作条件易变换,在手性分离上具有较优的前景。目前已有使用该技术对这3类毒品的对映异构体进行分离的研究^[60,61,62]^,还有将SFC与HPLC技术相结合的苯丙胺手性分离研究^[63]^,但该技术的缺点是各种参数对手性分离的影响机制尚未完全确定,需要在高压下进行,对设备和技术上要求较高,如果未来能解决上述问题,SFC技术可能成为对映异构体分离中常用的技术之一。

根据国家食品药品监督管理总局、公安部、国家卫生和计划生育委员会公布的《麻醉药品和精神药品品种目录》(食药监药化监[2013]230号)和公安部、国家卫生计生委、国家食品药品监督管理总局、国家禁毒办公室发布的《非药用类麻醉药品和精神药品列管办法》(公通字[2015]27号)及4次增加公告,截至目前,我国共管制麻醉药品121种,精神药品149种,非药用类麻醉药品和精神药品156种(另加156种以外其他符合条件的芬太尼类物质),而能对人体中枢神经系统造成严重危害的药品数量远远不止这些,未纳入管制的毒品中还包括一部分手性毒品。未来毒品对映异构体的研究将是毒品来源判断、纯度认定(掺杂、掺假)、对犯罪嫌疑人定罪量刑判断的一个重要方向。随着新精神活性物质种类的不断增加,毒品种类和成分越来越复杂,涉及的对映异构体分离检测会逐渐增多。今后对毒品手性分离的研究应从以下3个方面展开:(1)进一步借助计算机技术对手性固定相和手性选择剂的手性识别机理进行深入研究,建立和完善分子模型模拟手性识别方法,为日后挑选最合适的手性固定相和手性选择剂进行研究提供有力的理论支持;(2)研发可以应用于手性分离的新型技术,以及对SFC技术的商用研究;(3)手性分离在司法实践、医药研发等实际工作中的应用。
